# Toll-Like Receptor-1 and Receptor-2 and Beta-Defensin in Postcholecystectomy Bile Duct Injury

**DOI:** 10.1155/2015/216129

**Published:** 2015-02-08

**Authors:** Alejandra Guillermina Miranda-Díaz, José Manuel Hermosillo-Sandoval, Martha Arisbeth Villanueva-Pérez, Luis Miguel Román-Pintos, Trinidad García-Iglesias, Adolfo Daniel Rodríguez-Carrizalez, Ernesto Germán Cardona-Muñoz

**Affiliations:** University Health Sciences Centre, University of Guadalajara, Avenida La Paz No. 2758, Colonia Arcos Sur, 44150 Guadalajara, JAL, Mexico

## Abstract

Postcholecystectomy bile duct injuries (BDI) produce hepatic cholestasis and cause infection of the biliary tract. The biliary cells participate in secreting cytokines and in expression of immune response receptors. Toll-like receptors (TLRs) conduct signalling and activate the innate and adaptive inflammatory response. The objective was to determine the serum levels of TLR-2 and the expression of TLR-1 and TLR-2 and *β*-defensin in liver biopsies of postcholecystectomy BDI patients. A transverse, analytical study with 2 groups was done. One group included healthy volunteers (control group) and other included 25 postcholecystectomy BDI patients with complete biliary obstruction. Using the Enzyme-linked Immunosorbent Assay (ELISA) technique, serum levels of TLR-2 were determined, and with immunofluorescence the morphologic analysis of TLR-1 and TLR-2 and *β*-defensin in liver biopsies of postcholecystectomy BDI patients was performed. The average TLR-2 serum level in the control group was 0.0 pg/mL and in the BDI group, 0.023 ± 0.0045 pg/mL (*P* < 0.0001, bilateral Mann Whitney *U*). Immunofluorescence was used to determine the expression in liver biopsies, blood vessels, bile ducts, and hepatic parenchyma where 12 hepatic biopsies were positive for TLR-1 with average of 3213057.74 ± 1071019.25 *μ*m^2^; and 7 biopsies were positive for *β*-defensin with an average of 730364.33 ± 210838.02 *μ*m^2^; and 6 biopsies positive for TLR-2, obtaining an average of 3354364.24 ± 838591.06 *μ*m^2^. In conclusion, TLR-1 and TLR-2 and *β*-defensin play an important role in the innate antimicrobial defense of the hepatobiliary system.

## 1. Introduction

Postcholecystectomy bile duct injuries (BDI) are feared complications in biliary surgery because they cause stenosis of the bile ducts with disastrous consequences, which can lead to secondary biliary cirrhosis, hepatic insufficiency and, as an ultimate consequence, cause death [1].

Management of BDI is complicated, requiring experience, hepatobiliary capabilities, and a tertiary level of care [2]. Its prognosis is related directly to the individual condition of each patient, to the time between identification of the BDI and the efficacy of the biliary-digestive reconstruction [3].

Under normal conditions human bile is sterile. Acute cholangitis is the infection of the bile tract characterized by fever, right quadrant abdominal pain, and jaundice [4]. The etiology of cholangitis is diverse, among which are: choledocholithiasis, bile duct atresia in newborns, and complete obstruction of the bile ducts after cholecystectomy. For cholangitis to develop, biliary stasis with an increase in biliary pressure and the presence of microorganisms in the bile tracts must occur. The most commonly found pathogens are Gram-negative with the production of lipopolysaccharides (LPS) [5]. In the event that the cholangitis is not resolved rapidly, biliary obstruction unchains the development of sepsis and the systemic inflammatory response syndrome (SIRS), causing high morbidity and mortality with inadequate hepatic and systemic response in the presence of the LPS [6].

The biliary epithelial cells are immunologically powerful, and when inflammation in the bile ducts exists they participate in secreting cytokines and in expression of the immune receptors. In addition, the cells of the biliary epithelium recognize microbes through pattern recognition receptors (PRRs). The best microbe identifier is the pathogen associated molecular pattern (PAMP) via receptors such as the toll-like type (TLRs). When exposed to LPS the immune competent cells are activated and transmit intracellular adaptor signals associated to the TLRs, on favoring activation of the nuclear factor kappa-light-chain-enhancer of activated B cells (NF-*κ*B) and proinflammatory cytokines [7].

TLRs are expressed in immune cells including the polymorphonuclear (PMN) and epithelial cells. They have an extracellular domain with regions rich in leucine, one or two regions rich in cysteine, and an intracellular domain of approximately 200 amino acids through which they carry out the transmission of signals [8]. The ligands bind to the TLR to conduct signalling and activate the innate and adaptive immune response. The innate immune system induces phagocytosis, opsonization, and production of mediators of inflammation, blocking the dissemination of the pathogens. The TLRs that are expressed in the presenter cells of the antigen after recognizing the ligands are activated and induce molecules that participate in the presentation of antigenic peptides on the surface. In the major histocompatibility complex the peptides are recognized by the antigen specific T cells, uniting, in that way, the innate and adaptive immune response [9].

The activation and expression of the TLR-1 and TLR-2 and *β*-defensin have not been sufficiently analysed. For that reason, the study proposed to determine the serum levels of TLR-2 and, through immunofluorescence, evaluate the expression of the TLR-1 and TLR-2 and *β*-defensin in liver biopsies of postcholecystectomy BDI patients.

## 2. Material and Methods

A transverse, analytical study using two formed study groups was done.

*Group  1*. Healthy volunteers served as a control group. 5 mL of peripheral blood was withdrawn; the serum was separated and stored at −80°C until processing.

*Group  2*. It included 25 postcholecystectomy BDI patients with complete biliary obstruction who were sent to the Department of General Surgery at the Specialties Hospital of the National Occidental Medical Health Centre of the Mexican Social Security Institute as a tertiary care facility to undergo the most appropriate biliary-digestive reconstruction depending on the individual characteristics of each patient and their type of biliary lesion. Prior to the administration of anaesthesia, 5 mL of peripheral blood was withdrawn; the serum was separated and stored at −80°C until processing. Because hepatic biopsy and histopathologic examination are routinely performed on all patients subjected to bile duct reconstruction, the immunofluorescence was performed on a portion of those biopsies.

### 2.1. ELISA TLR-2

A commercial ELISA test was used for the study (R&D Systems Duo-Set, Category number DY2616). The procedure was performed in duplicate according to the manufacturer's instructions. The curve pattern of 7 points was prepared; 18 *μ*L of the diluent was pipetted into the tubes of 8000 pg/mL and 100 *μ*L of the standard; 500 *μ*L of the calibrator diluent was pipetted into the tubes, and 500 *μ*L of the standard was added before the serial dilutions 1 : 2. The undiluted standard served as the highest standard and the calibrator diluent as the zero. 100 *μ*L of the diluent was added, and then 100 *μ*L of the sample was placed in each well. It was incubated for 2 hours at room temperature and then washed and the buffer was removed in turn; 100 *μ*L of the detecting antibody was added, and it was incubated for 2 hours. Other washings were performed and 100 *μ*L of the Streptavidin-HRP solution was added and incubated for 20 minutes at room temperature, and then 50 *μ*L of the stop solution was added. The optical density was measured at 450 nm.

### 2.2. Immunofluorescence of TLR-1 and TLR-2 and *β*-Defensin

The manufacturer's instructions were followed (Santa Cruz Biotechnology USA, catalogue sc 3000, sc 10739, and sc 10860, USA). First, the antibodies were diluted with PBS to a ratio of 1 : 50 to be considered positive. On electrically charged lamellae the cryostatic, frozen cuts of 5 microns were made; the samples are placed in acetone for 10 minutes, and let them dry for about 25 minutes; afterwards, they were immersed in PBS buffer for 10 minutes without agitation, in a dark, humid chamber, and one drop (the equivalent of 25 *μ*L) of the antibody of interest was added to each of lamellae. They were then incubated in a humid chamber, protected from light, at room temperature, for 1 hour. After incubating, the lamellae were electronically agitated to eliminate the excess of antibodies and then washed, immersed in a PBS buffer, for 10 minutes, without agitation, and protected from light and exposure to air. The plates were removed, the excess PBS buffer was eliminated, and they were covered with aqueous mounting medium, protected from light. The fluorescence was established on observing the samples with a fluorescence microscope with a wavelength of 495 nm and filter of 515 nm. The images were taken at 10x magnification with an AxioCam high resolution camera (Carl Zeiss). On the laminates random fields were measured, and the 4.6.3 software (Carl Zeiss) was used to perform the morphometric analysis in *μ*m^2^.

### 2.3. Statistical Analysis

Statistical analysis was performed with SPSS software. Quantitative evaluations were expressed as mean ± standard error, and qualitative variables were expressed as frequencies and percentages. Data did not follow a normal distribution (Shapiro-Wilk test), and comparisons were analyzed using a Mann Whitney *U* test. A two-tailed value of *P* ≤ 0.05 was considered significant. The confidence interval was 95%.

## 3. Results

### 3.1. Clinical Characteristics

21 females and 4 males with postcholecystectomy BDI were included. The majority of those included were at productive life stages, with the average age being 46.6 years; 6 patients were >60 years of age.

The most frequent type of lesion was Bismuth III (10), Bismuth II (9), Bismuth I (5), and Bismuth IV (1) [10]. The injury was identified during cholecystectomy in 7 patients and was handled with placement of external biliary drainage and referral to tertiary-care hospital.

The elective, surgical reconstruction was performed 6 weeks after lesion. Preoperatively, patients were subjected to artificial nutrition and external biliary drainage. Their coagulation tests were normalized in order to minimize the risk of intraoperative bleeding. In the surgical room whole blood, fresh frozen plasma and vitamin K were provided.

The biliary-digestive reconstructions predominantly included the Hepp-Couinaud or Y de Roux, and the average length of surgery was 6 hours. On hospitalization, all patients presented with jaundice and right quadrant pain. One patient also presented with surgical wound infection, 1 presented with free bile material in the abdominal cavity, and 2 presented with cholangiolar abscesses.  Table 1 shows the liver enzymes alanine aminotransferase (ALT), aspartate aminotransferase (AST), alkaline phosphatase (AP), and direct bilirubin (DB) before surgical reconstruction. The enzymes were increased before the surgical reconstruction but after the percutaneous drainage without statistical significance versus healthy control group.

### 3.2. ELISA TLR-2 and Immunofluorescence of TLR-1 and TLR-2 and *β*-Defensin

The result of the curve pattern in the ELISA test was an *R* ^2^ value of 0.944. The average TLR-2 blood serum level in the control group was 0.0 pg/mL, and in BDI 0.023 ± 0.0045 pg/mL (*P* < 0.0001 bilateral Mann Whitney *U*) (Figure 2).

Of the 25 patients included in the study who were positive for TLR-2 in the ELISA test, 12 hepatic biopsies were positive for TLR-1 and the morphometric analysis of 141 random fields showed average of 3213057.74 ± 1071019.25 *μ*m^2^; in the 7 biopsies positive for *β*-defensin, 135 random fields were measured with an average of 730364.33 ± 210838.02 *μ*m^2^ and in 6 biopsies positive for TLR-2, 190 random fields were measured obtaining an average of 3354364.24 ± 838591.06 *μ*m^2^. (Figure 3).

**Figure 1:**
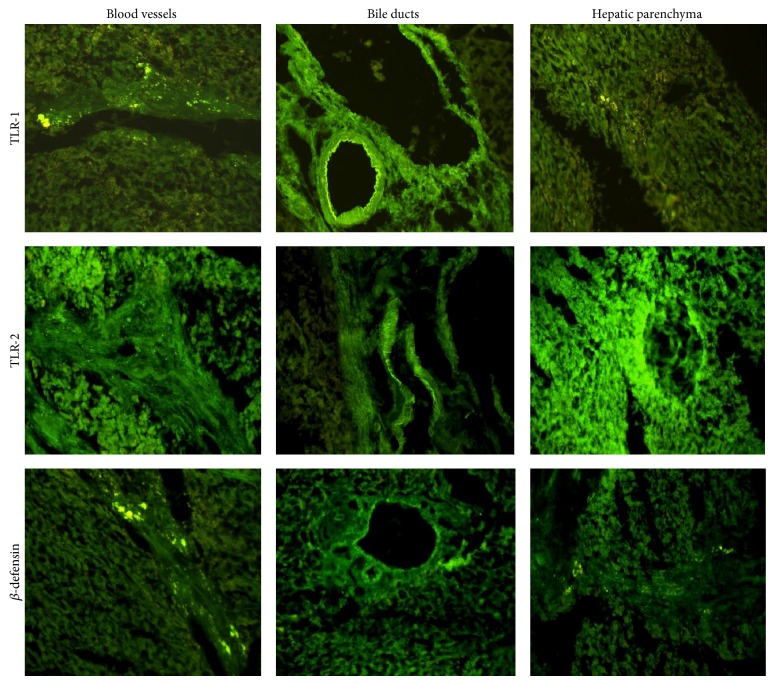
Immunofluorescence of TLR-1 and TLR-2 and *β*-defensin in postcholecystectomy BDI. The antibodies anti-TLR-1 and TLR-2 and *β*-defensin can be positively observed in blood vessels, bile ducts, and hepatic parenchyma of patients who suffered postcholecystectomy BDI. In the immunofluorescence of the epithelium of the bile ducts in liver biopsies, the higher expression of TLR-1 and TLR-2 is attention grabbing, although *β*-defensin is also seen.

**Figure 2:**
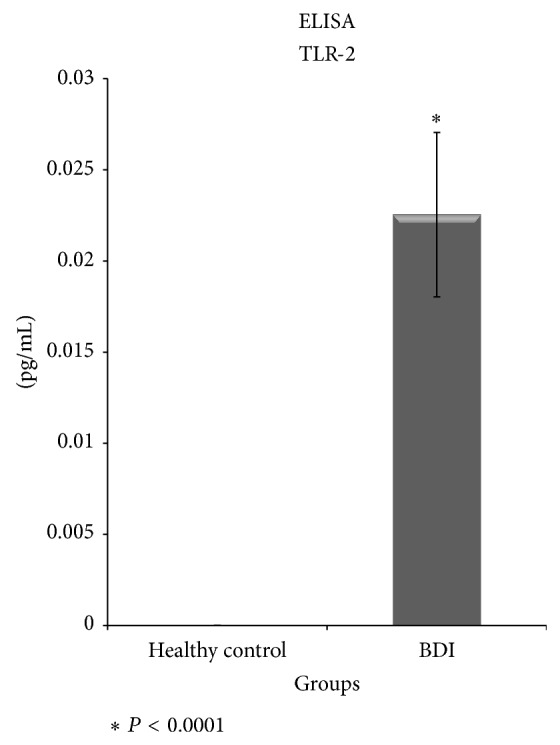
ELISA of TLR-2 IN postcholecystectomy BDI. In the control group it was not possible to detect levels of TLR-2; however, in BDI levels of TLR-2 were significantly detected, suggesting an innate and adaptive response to bacterial colonization.

**Figure 3:**
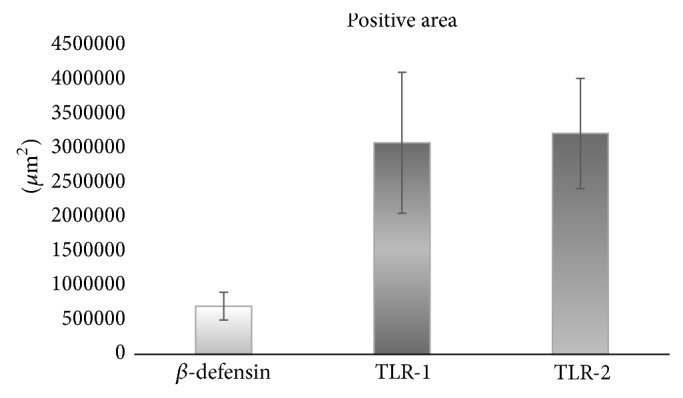
Morphometric analysis of the liver biopsies. The average ± standard error in the quantification of positive areas in the liver biopsies of patients with bile duct injuries subjected to biliary-digestive reconstruction can be obtained. The higher expression of TLR-1 and TLR-2 than *β*-defensin can be appreciated, which suggests deregulation between the innate and adaptive immune responses when faced with infection of the bile pathways.

Figure 1 shows the blood vessels, bile ducts and hepatic parenchyma in the most representative images from the biopsies subjected to immunofluorescence, positive results for the antibodies TLR-1, TLR-2, and *β*-Defensin.

## 4. Discussion

Lipopolysaccharides are proteins of Gram-negative bacteria with the ability to initiate the cascade of signal transmission that conducts the liberation of inflammatory cytokines. However, Gram-positive bacteria also mediate the induction of inflammatory cytokines through the liberation of lipoteichoic acid and peptidoglycan fragments. On different occasions it has been demonstrated that these substances act as powerful cytokine inductors, interacting with the TLR-2, in synergy with the TLR-1 or TLR-6 [11]. The infected cells recognize components of the cell wall (lipoteichoic acid) of Gram-positive bacteria by way of the CD14 and TLR-2 receptors. When the lipoteichoic acid binds with the TLR-2 it induces activation of the mechanisms of transmission and secretion of proinflammatory cytokines such as interleukin-1*β*, interleukin-6, and the tumour necrosis factor TNF-*α*. The TLRs are the primary sensors of the innate and adaptive immune response, responsible for recognizing PAMPs.

The TLR-2 recognizes a wide range of bacterial products through cooperation with diverse proteins. The TLR-2 forms heterodimers with TLR-1 and TLR-6 that are structurally related. The TLR-2 collaborates with distinct types of receptors of components of the cell wall of fungi (dectin-1). The cytoplasm domains of all TLRs are not functionally equivalent, which suggests that the capacity of different cellular specific types to respond to Gram-positive bacteria is not defined solely by expression of the TLR-2. The differential expression and heterodimerization between the TLRs increase the repertoire of cellular responses activated by diverse infectious stimuli as a specific cellular response. In our study, of the 25 patients with ELISA results that were positive for TLR-2, only 6 hepatic biopsies were positive for the antibody anti-TLR-2, suggesting that the systemic response was detectable and that the hepatic expression was controlled by the biliary epithelium or bound to the TLR-1 forming heterodimers. Since the TLR-2 is liberated as a response to infections by Gram-positive or Gram-negative bacteria, we can consider that BDI patients who responded positively in the ELISA for TLR-2 possibly did so because only patients with complete biliary obstruction were included in the study and all presented with, to greater or lesser degrees, acute cholangitis. During the surgical reconstruction purulent material, concentrated bile and/or fibrin deposits, was frequently found free in the abdominal cavity, which could have contributed to the systemic response to the infection. The TLR-2 serum levels in BDI could be related to the rapid systemic effect present in postcholecystectomy BDI. However, the systemic response contrasts with the expression of TLR-2 in only 6 hepatic biopsies. This response could relate to the hepatic expression of human *β*-defensin in 7 biopsies, possibly due to the clearing of the biliary epithelium or by alteration of the innate and adaptive immune response in postcholecystectomy BDI by the *β*-defensin. The defensins are antimicrobial peptides, key to innate immunity. Structurally, they are divided into *α*- and *β*-defensins and are distributed in the epithelium of various organs in forming a barrier on the mucosal surfaces. Numbers 1–6 of human *β*-defensins have been identified; they are innately expressed in cultivated biliary endothelial cells and are distributed in the cytoplasm of the intrahepatic bile ducts. It seems that they play an important role in the innate antimicrobial defense of the hepatobiliary system. Normally, infections of the bile ducts are rare in physiological conditions, although the biliary tree is potentially exposed to enteric bacteria. It has been reported that human *β*-defensin is not detected in cultivated biliary epithelial cells without the addition of stimulants (LPS or* Escherichia coli*). The expression of human *β*-defensin-2* in vivo *is seemingly restricted to the large intrahepatic ducts and in the peribiliary glands, in the presence of acute cholangitis, and in extrahepatic biliary obstruction of different etiologies. The participation of bacteria related to cholangitis is widely associated with the expression of human *β*-defensin-2 in cells of the biliary epithelium. The human *β*-defensin-1 plays a fundamental role in the biliary antimicrobial defense, while the induction of the expression of human *β*-defensin-2 is present in response to infections [12].

The TLR-1, also known as CD281, is expressed in monocytes/macrophages, dendritic cells, granulocytes, and some lymphocytes. The TLR-1, in association with TLR-2, functions as a coreceptor implicated in response to diverse pathogenic agents, producers of triacylated lipopeptides, peptidoglycans, and lipoteichoic acid. In our study, 12 hepatic biopsies were positive for TLR-1 in the blood vessels, bile ducts, and hepatic parenchyma. The epithelium of the bile pathways is exposed to microbes and their products because the majority of hepatic circulation stems from portal circulation that recollects venous blood from the intestine; therefore, it is not strange to find an inflammatory and immunological response in the bile tract. Nevertheless, the cytokines secreted by the cholangiocytes, in conjunction with the molecules of adhesion and the TLRs expressed in their surface, play an important role in recruitment, localization, and regulation of the immune response in liver and the bile pathways [13].

The innate biliary immunity is important for immunity of the epithelium of the intra and extrahepatic bile ducts, is associated with the pathogenesis of diverse biliary illnesses, and could be molecular targets in the defense against microbial infections. In conclusion, determining the behaviour of TLRs in postcholecystectomy BDI seems useful for proposing potential, new therapeutic strategies.

## Figures and Tables

**Table 1:** Liver enzymes. Serum levels of liver enzymes were increased before the biliary digestive reconstruction in BDI patients without significant difference versus healthy controls.

	Normal	BDI	*P *
ALT (UI/L)	14.75 ± 7.38	200.50 ± 53.59	NS
AST (UI/L)	16.50 ± 8.25	118.15 ± 32.77	NS
AP (UI/L)	52.75 ± 26.38	472.86 ± 126.38	NS
DB (mg/dL)	0.30 ± 0.15	4.35 ± 1.21	NS
